# Interpersonal and socio-demographic factors associated with divorce among women of reproductive age in Tanzania: evidence from the 2015-16 Tanzania Demographic and Health Survey data

**DOI:** 10.11604/pamj.2026.53.18.42894

**Published:** 2026-01-15

**Authors:** Idda Hubert Mosha

**Affiliations:** 1 Department of Behavioural Sciences, School of Public Health and Social Sciences, Muhimbili University of Health and Allied Sciences, Dar es Salaam, Tanzania

**Keywords:** Divorce, separated, women of reproductive age, interpersonal factors, socio-demographic factors

## Abstract

**Introduction:**

divorce is a global issue affecting communities worldwide and is linked to adverse social outcomes such as school dropouts and an increase in street children. This study aimed to examine socio-demographic and interpersonal factors associated with divorce among women of reproductive age in Tanzania.

**Methods:**

a cross-sectional analysis was conducted using data from the 2015-16 Tanzania Demographic and Health Survey and Malaria Indicator Survey (TDHS-MIS), a nationally representative survey. The study population included women aged 15-49 years who had ever been married or cohabited. Multivariable logistic regression was used to identify factors associated with divorce.

**Results:**

a total of 9,443 women were included, with a mean age of 31.5 ± 8.7 years; 13.3% were divorced or separated. Early sexual debut, particularly before age 15, was associated with higher odds of divorce (AOR 1.52, 95% CI: 1.20-1.91, p<0.001). Employment increased the likelihood of divorce across all occupational categories compared with domestic work. Other socio-demographic factors linked to divorce included urban residence (AOR 1.32, 95% CI: 1.06-1.63), mobile phone ownership (AOR 1.73, 95% CI: 1.45-2.08), and having a partner who consumed alcohol (AOR 1.46, 95% CI: 1.24-1.71). Interpersonal factors such as fear of the husband, accusations of infidelity, and restrictions on social or family contacts also increased the odds of divorce.

**Conclusion:** divorce among women of reproductive age in Tanzania is influenced by multiple factors. Interventions such as family life education and premarital counseling are recommended to promote harmonious relationships and reduce the risk of divorce.

## Introduction

Marriage is an experience that the majority of people worldwide go through [1]. In contemporary society, divorce has become increasingly common, impacting many families across the globe. There is an increase in divorce rates in Europe. For instance, the divorce rate in Great Britain rose by 9.8% in 2005 [2]. Additionally, in 2008, 30% of single-parent families in Britain and 20% in France were a result of divorce [3,4]. The incidence of divorce has grown across the globe [5]. Research in 33 sub-Saharan African countries revealed that one-quarter of first unions ended in divorce, ranging from 6.9% in Mali to 47.1% in Congo [6]. Marriage in Africa is considered a bond between a man and a woman with the purpose of procreation and is widely acknowledged as a social and legal institution [7]. Marriage is the least restrictive way society uses to safeguard the well-being of children [1,8]. Marriage is a social institution connected to various other systems, including economic, cultural, and political structures [9]. However, some marriages ultimately result in divorce. Divorce represents the formal legal dissolution of a marriage, and those affected may be permitted to marry again according to civil, religious, or other regulations, depending on the laws of the respective country [8]. Divorce is often regarded as a recourse in response to abusive or dysfunctional marital relationships [10]. The consequences of divorce may produce both positive and negative effects for the parties involved [11]. Divorce is associated with various socio-economic and health challenges for both the divorced individuals and their children [12,10]. Research from developed countries has demonstrated that children of divorced parents experience greater mental and physical health challenges compared to children of married parents [13]. The impact of divorce on children in developing regions appears to be more pronounced than in high-income regions [14]. Divorce has been associated with a range of adverse outcomes in children, including behavioral and emotional difficulties, heightened stress, exposure to violence, compromised parenting, substance abuse, increased prevalence of street children, limited access to education and essential services, and impaired relational skills [11,15]. Divorce may also result in adverse outcomes, particularly for children, who often experience emotional distress and confusion stemming from the separation of their parents [16]. Divorce can elicit feelings of guilt, confusion, and sadness in children, potentially resulting in long-term psychological and emotional impacts. Studies in sub-Saharan Africa (SSA) reveal a heightened mortality risk among children of divorced parents. In particular, research from Ethiopia and Malawi shows that children whose mothers remarried were at greater risk of death relative to those whose mothers maintained continuous marital unions [17].

A study from another African country reported that children of divorced parents exhibited a higher risk of under-five mortality. Among the surviving children, there was an increased likelihood of stunting, delayed school enrollment, and lower educational attainment compared to their peers from continuously married households. Furthermore, divorce can entail substantial economic consequences, particularly for women, who are often more susceptible to financial instability and poverty following marital dissolution [11]. The economic ramifications of divorce can be particularly severe for women. Following divorce, children frequently reside with their mothers, and the loss of the former husband´s income often results in a significant decline in household financial resources [18]. For example, it was also revealed that divorce exacerbates poverty inequalities, principally due to the higher risk and greater vulnerability of individuals with lower levels of education [19]. Several studies have further documented that women often experience economic disadvantage following divorce [11]. For example, research has shown that divorce lowers women's salaries [20,21]. This demonstrates how women's living standards decline and they become more vulnerable to poverty as a result of divorce-related income reductions. As a result, following a divorce, women experience significant financial difficulties. In addition, dividing assets, selling houses, sharing debts, and child custody can be challenging for separating spouses. Divorce is brought on by a wide range of complicated problems that are frequently interrelated and multifaceted. Divorce is frequently caused by unfaithfulness or infidelity, which can lead to emotions of betrayal, mistrust, and communication failures in a partnership [22]. Incompatible differences, disagreements over parenting, and financial difficulties are other common causes of divorce. Certain marriages may prove to be irreparable, even though some couples may try to resolve these problems through therapy. Social media use has also been connected to divorce [23] partner education levels, and gender-based violence [24]. Nonmarital cohabitation has become a frequent element of young people's life, the percentage of people who never marry is increasing, and cohabitation is probably going to grow more widespread as divorced people choose to live together rather than get married again [25]. According to certain research, factors that predicted divorce included age at marriage, employment position, partner abuse, globalization, sexual pleasure, and financial difficulties [6,26,27].

According to some research, one of the causes of divorce is infertility [28] considering the cultural norms and societal expectations around marriage in the African environment. According to a different survey, procreation is a top goal for African males in marriage, and having a child is seen as essential to starting a long-lasting family [29]. Additionally, Adedokun [30] claimed that the frequency of divorce and separation increased with educational attainment. Similarly, it has been shown that divorce is linked to early marriage and difficult financial situations [31]. Divorce is often caused by abuse, alcoholism, infidelity, financial difficulties, abrupt character changes, and poor communication [22]. According to a different Ethiopian study, women who had experienced relationship violence were more likely than their peers to get divorced [24]. Similarly, it was found that young couples who were subjected to physical and sexual abuse were more likely to file for divorce [32]. Although it has been said that adultery, substance abuse, domestic violence, and lack of commitment are the leading causes of divorce [33]. Divorce rates are considerably higher for women who experience abuse, which can be brought on by things like alcoholism and violence from male partners [32]. It was discovered that accessing social media is linked to divorce. It was also discovered that excessive reliance on social media fosters skepticism, envy, and mistrust amongst couples, straining idealized relationships [34]. Similarly, it has been argued that utilizing social networking sites is inversely associated with marriage quality and happiness, but positively associated with a difficult relationship and divorce [35]. Social media can contribute to infidelity and divorce [23]. The study also found that social media use can negatively impact relationships and lead to divorce. Broadband internet prevalence and use have had a substantial impact on divorce rates, which are greater in places with lower education levels and higher income growth rates [36]. Similarly, in Iran, it was discovered that there is a positive association between the mobile diffusion rate and the divorce rate over both short and long periods [37].

It has been observed that women with no education or who had attended a literacy program had a reduced probability of divorce than women with primary education. Furthermore, women with secondary and higher education are 0.63 times more likely to divorce than women with primary education [38]. The association between educational attainment and divorce is equivocal, with some studies showing a positive correlation and others showing a negative one. Highly educated women who earn more than their husbands are more likely to exit poor marriages while staying in excellent ones [39]. Similarly, a study conducted in Ethiopia discovered that completing secondary education doubled the likelihood of marriage breakdown compared to having no formal education [40]. Data from Demographic and Health Surveys from 33 SSA nations reveal that urbanization and female employment were correlated with greater levels of divorce, while age at first marriage and better female education were associated with lower rates of divorce [6]. There is also a link between divorce and education; women with poor education were more likely to experience divorce [24]. Tanzanian law supports divorce by allowing any married person to petition the court for a decree of separation or divorce because their marriage is illegal, and divorced people have the right to remarry and collect maintenance from their ex-spouses under any orders [11]. In recent years, Tanzania has seen an increase in divorce lawsuits. According to media sources, divorce is becoming more common in cities. According to the National Bureau of Statistics' (NBS) report on the National Panel Survey of 2014/15, the divorce rate has doubled in the last six years [41]. For example, it has been established that in Dar es Salaam alone, at least 360 marriages are breaking apart each month - an average of 12 per day [42-44]. This is an uncommon phenomenon in African societies, affecting both family stability and employment. Likewise, it was stated that in Tanzania, 10.4% of women and 5% of males are divorced [44]. Rising divorce rates in Tanzania are a source of concern since they may have harmful implications for the individuals involved and their children. These repercussions might be both short-term and long-term. Divorce has a significant impact on the psychological, socioeconomic, emotional, and physical well-being of millions of children, adolescents, and adults [45]. Families disintegrate, resulting in a variety of social and psychological issues. Divorce cases also inhibit development in the country since family units, which form the functional core of society, disintegrate, causing moral underpinnings to be lost and destroying vision for the future generation. Divorce disrupts family stability and the proper operation of the workplace; individuals may also suffer from poverty as a result of the divorce. Given Tanzania's escalating divorce rates and the potentially far-reaching effects for people and families, it is critical to get a greater understanding of the factors associated with divorce among women of reproductive age in Tanzania. The purpose of this study was to examine interpersonal and socio-demographic factors associated with divorce among women in Tanzania, in order to help various marriage stakeholders in Tanzania find solutions to this developing issue. Furthermore, the outcomes of this study can assist design various preventive strategies for lowering divorce rates.

## Methods

**Study design and data source:** this was a cross-sectional study using the TDHS-MIS dataset from 2015-16. From August 2015 to February 2016, the TDHS-MIS collected data on mother and child health, reproductive health, nutrition, and other health subjects [46].

**Study setting:** the 2015-16 TDHS-MIS is a nationwide cross-sectional survey that measures levels, patterns, and trends in demographic and health indicators [46]. The 2015-16 TDHS-MIS gathered data on fertility levels, marriage, sexual activity, fertility desires, family planning awareness and use, breastfeeding habits, nutrition, childhood and maternal mortality, maternal and child health, malaria, and other health-related topics. The sample design for the 2015-16 TDHS-MIS was completed in two stages and was designed to produce estimates for the entire country, urban and rural areas on Tanzania's mainland, and Zanzibar. The initial stage entailed choosing clusters. These were the enumeration regions for Tanzania's 2012 Population and Housing Census. A total of 608 clusters were identified. In the second step, a methodical selection of 13,360 households was made, and 12,767 of those households were inhabited. Of the occupied households, 12,563 were successfully interviewed, producing a 98% response rate. In the questioned households, 13,634 suitable women were identified for individual interviews; 13,266 women were interviewed, resulting in a 97% response rate.

**Study population and sample size:** the study included women of reproductive age (15-49 years) who had previously married or cohabited. Data on eligible women were retrieved from the individual recode file (TZIR7BFL). This analysis excludes persons who reported never being married, as there is no likelihood of divorce by the time of the survey. Thirteen thousand two hundred and sixty-six (13,266) women were interviewed and those who had never married during the survey period were excluded, leaving 9,443 eligible women for this study.

**Sampling technique:** the 2015-16 TDHS-MIS [46] used stratified two-stage cluster sampling. The first stage involved selecting clusters containing enumeration areas, from which 608 clusters were selected. The second stage entailed the systematic selection of households from the 608 identified clusters, with 22 households chosen in each cluster. This sampling technique provided a probability sample of 13,376 houses, of which only 12,767 were occupied. A total of 13,266 women were interviewed, with 9,443 of them included in this study.

**Measurement of variables:** the outcome variable (divorce/separation) was measured using a query on the present marital status. The independent variables included socio-demographic characteristics (age, level of education, place of residence, occupation, wealth index status, and parity) as well as interpersonal and structural factors such as mobile phone ownership, membership in VICOBA, and husband/partner-related variables.

**Data analysis:** the data were retrieved, cleaned, and analyzed by STATA version 18 (STATA Corp, College Station, Texas, USA). First, descriptive statistical analysis was performed on socio-demographic, interpersonal, and structural components to get frequencies and proportions for categorical variables, as well as means and standard deviations for continuous variables. The variables for the multivariable logistic regression model were then identified using univariate logistic regression. All explanatory factors with a P-value < 0.2 on univariate analysis were submitted to multivariable logistic regression to further analyze the association and generate adjusted odds ratios. To account for the changes in sampling probabilities between clusters and strata, I employed sample weighting to compensate for the cluster sampling design.

**Ethical considerations:** this study used secondary data and did not involve any human volunteers. Thus, no official ethical approval was necessary. Nevertheless, the Tanzania Demographic and Health Survey was carried out with the approval of national and international review boards such as the National Institute of Medical Research, Zanzibar Medical Research Ethical Committee, Inner City Fund Institutional Review Board, and the Centers for Disease Control and Prevention in Atlanta. Before the study began, all of the women interviewed were asked to provide verbal informed consent. The clearance to utilize the IPTp-SP data was secured from the DHS program.

## Results

**Socio-demographic characteristics:** the analysis comprised 9,443 women, with an average age of 31.5 ± 8.7 years. Table 1 shows the distribution of socio-demographic factors. Out of 6,333 participants, 66.4% had a primary education level. Of all the participants, 93.1% had at least one child and 67.8% lived in rural areas (Table 1)

**Table 1:** distribution of socio-demographic characteristics

Variables	Unweighted: N (%)	Weighted: N (%)
**Age groups: Mean ± SD**	31.64 ± 8.72	31.46 ± 8.72
15-19	684 (7.2%)	734[ 7.7%)
20-29	3,517 (37.2%)	3,583 (37.6%)
30-39	3,117 (33.0%)	3,118 (32.7%)
40-49	2,125(22.5%)	2,098 (22.0%)
**Education level**		
No education	1,809 (19.2%)	1,773 (18.6%)
Primary	5,885 (62.3%)	6,333 (66.4%)
Secondary+	1,749 (18.5%)	1,428 (15.0%)
**Age at first sex Mean±SD**	16.97 ± 2.88	16.80 ± 2.68
Below 15	1,403 (14.9%)	1,425 (14.9%)
15-17	4,587 b(48.6%)	4,838 (50.7%)
18+	3,453 (36.6%)	3,270 (34.3%)
**Age at first marriage/cohabitation: Mean ± SD**	18.76 ± 3.87	18.76 ± 3.90
Below 15	759 (8.0%)	767 (8.0%)
15-17	3,203 (33.9%)	3,261 (34.2%)
18+	5,481 (58.0%)	5,506 (57.7%)
**Duration since first marriage: Mean ± SD**	12.699 ± 8.843	12.874 ± 8.902
0-4	2,127 (22.5%)	2,187 (22.9%)
5-9	1,852 (19.6%)	1,891 (19.8%)
10+	5,464 (57.9%)	5,455 (57.2%)
**Parity**	3.66± 2.64	3.52± 2.57
No child	636 (6.7%)	658 (6.9%)
Had at least one child	8,807 (93.3%)	8,875 (93.1%)
**Type of place of residence**		
Urban	2,618 (27.7%)	3,070 (32.2%)
Rural	6,825 (72.3%)	6,464 (67.8%)
**Occupation**		
Not working	1,488 (15.8%)	1,440 (15.1%)
Professional	691 (7.3%)	747 (7.8%)
Agricultural	4,907 (52.0%)	4,898 (51.4%)
Household and domestic	374 (4.0%)	442 (4.6%)
Manual works	1,983 (21.0%)	2,007 (21.0%)
**Wealth index status**		
Poorest	1,770 (18.7%)	1,862 (19.5%)
Poor	1,695 (17.9%)	1,785 (18.7%)
Middle	1,842 (19.5%)	1,782 (18.7%)
Rich	2,116[ 22.4%)	1,932 (20.3%)
Richest	2,020 (21.4%)	2,173 (22.8%)

**Interpersonal and structural factors:** more than half of the participants, 5,119 (53.7%), had a cell phone. Only 986 participants (10.3%) used VICOBA to conduct financial transactions. The majority of participants, 4,442 (59.4%), said that their husbands feel envious when they chat to other guys, and their husbands demanded to know where the responder was, 4,236 (56.7%).

**Distribution of divorce by socio-demographic characteristics:** women were asked about their marital status in the poll. The findings indicate that 1,254 (13.3%) of women were divorced or separated. Table 2 displays the findings of a bivariate study of divorce and sociodemographic factors. Divorce was linked with older age, younger age at first sex, longer marriage duration, having at least one kid, living in an urban location, and occupation (Table 2).

**Table 2:** bivariate results for socio-demographic characteristics associated with divorce

Variable	Divorced n (%)	OR (95% CI)	P-value
**Age groups**			
15-19 (ref)	65 (9.50)	Ref	Ref
20-29	454 (12.91)	1.41 (1.07 -1.86)	.014
30-39	411 (13.19)	1.45 (1.10-1.91)	.009
40-49	324 (15.25)	1.71 (1.29-2.27)	<.001
**Education level**			
Below secondary	1036 (13.46)	Ref	Ref.
Secondary+	216 (12.46)	0.92 (0.78-1.07)	.266
**Age at first sex**			
Below 15	229 (16.32)	1.45 (1.22-1.72)	<.001
15-17	615 (13.41)	1.15 (1.00-1.31)	.041
18+ (ref)	410 (11.87)	Ref.	Ref.
**Age at first marriage**			
Below 15	110 (14.49)	1.09 (0.88-1.35)	.439
15-17	406 (12.68)	0.93 (0.82-1.06)	.294
18+ (ref)	738 (13.46)	Ref.	Ref.
**Duration since first marriage**			
0-4 (ref)	231(10.86)	Ref	Ref.
5-9	268 (14.47)	1.39 (1.15-1.68)	<.001
10+	755 (13.82)	1.32 (1.12-1.54)	<.001
**Parity**			
No child (ref)	65 (10.22)	Ref.	Ref.
Had at least 1 child	1189 (13.50)	1.37 (1.05-1.78)	.019
**Type of place of residence**			
Urban	435 (16.61)	1.46(1.29-1.66)	<.001
Rural (ref)	819 (12.00)	Ref.	Ref.
**Occupation**			
Not working (ref)	100 (6.7)	Ref.	Ref.
Professional	91 (13.17)	2.10 (1.56-2.84)	<.001
Agricultural	569 (11.60)	1.83 (1.46-2.27)	<.001
Household and domestic	111 (29.68)	5.86 (4.34-7.91)	<.001
Manual	383 (19.31)	3.32 (2.63-4.19)	<.001
**Wealth index status**			
Poorest (Ref)	183 (10.34)	Ref.	Ref.
Poor	240 (14.16)	1.43 (1.16-1.76)	
Middle	256 (13.9)	1.40 (1.14-1.71)	
Rich	281(13.28)	1.33 (1.09-1.62)	
Richest	294(14.55)	1.48 (1.21-180)	

**Distribution of divorce by interpersonal and structural factors:** Table 3 presents the findings from the bivariate study of divorce, as well as the interpersonal and structural components. Owning a mobile phone, husband/partner drinking alcohol, being afraid most of the time, husband/partner being jealous, being accused by husband/partner of unfaithfulness, being restricted by husband/partner to meet female friends, being restricted by husband/partner to contact family, and husband/partner insisting on knowing where the respondent was where all factors associated with divorce/separation.

**Table 3:** bivariate results for interpersonal factors associated with divorce

Variable	Divorced n (%)	OR 95% CI)	P-value
Own a mobile telephone	789(15.44)	1.52(1.34-1.72)	<.0001
Use VICOBA for financial transaction	121 (1236)	0.91 (0.75-1.11)	.370
Husband/partner drinks alcohol	400 (17.63)	2.07 (1.79-2.39)	<.001
Respondent afraid of husband most of the time	313 (29.20)	4.19 (3.58-4.90)	<.001
Husband jealous if respondent talks with other men	625 (14.62)	1.93 (1.65-2.25)	<.001
Husband accuses partner of unfaithfulness	425 (20.21)	2.69 (2.34-3.11)	<.001
Husband/partner does not permit respondent to meet female friends	315 (27.16)	3.74 (3.20-4.37)	<.001
Husband/partner tries to limit respondent contact with family	208 (26.94)	3.27 (2.73-3.90)	<.001
Husband /partner insists on knowing where respondent is	592 (14.91)	1.91(1.65-2.22)	<.001

**Independent factors associated with divorce/separation:** Table 4 shows the multivariable logistic regression results for divorce/separation among women aged 15-49 in Tanzania. Divorce/separation was independently linked with lower age at first sex encounter (AOR 1.52, 95% CI 1.20-1.91, p<.001) for those who had first sex before 15 years compared to those who had first sex at 18 years or older. Women working in domestic or household occupations (AOR 5.31, 95% CI 3.64-7.76, p<.001), manual occupations (AOR 2.78, 95% CI 2.08-3.72, p<.001), professional occupations (AOR 2.17, 95% CI 1.48-3.18, p<.001), and agriculture (AOR 1.39, 95% CI 1.04-1.85, p=.024) had higher odds of divorce compared to those not working (AOR 1.39, 95% CI 1.04-1.85, p=.024). Divorce rates were greater among urban women (AOR 1.32, 95% CI 1.06-1.63, p=.012), those with mobile phones (AOR 1.73, 95% CI 1.45-2.08, p<.001), and those whose partner drank alcohol (AOR 1.46, 95% CI 1.24-1.71, p<.001). Other factors independently associated with higher odds of divorce were respondent's fear of husband (AOR 3.16, 95% CI 2.64-3.79, p<.001), husband/partner accusing respondent of unfaithfulness (AOR 1.58, 95% CI 1.31-1.89, p<.001), husband/partner not allowing respondent to meet female friends (AOR 2.04, 95% CI 1.66-2.50, p<.001), and husband/partner attempting to limit respondent contact with family (AOR 1.26, 95% CI 1.01-1.58, p=.044).

**Table 4:** multivariable logistic regression results for factors associated with divorce

Variable	AOR (95% CI)	P-value
**Respondent's current age**		
15-19	Ref.	Ref.
20-29	0.87 (0.58-1.32)	.525
30-39	0.95 (0.59-1.52)	.820
40-49	1.42 (0.87-2.32)	.158
**Age at first sex**		
Below 15	1.52 (1.20-1.91)	<.001
15-17	1.10 (0.92-1.31)	.286
18+(ref)	Ref.	Ref.
**Duration in marriage/cohabitation**		
Below 5 (Ref)	Ref.	Ref.
5-9	1.04 (0.79-1.37)	.798
10 and above	0.94 (0.69-1.27)	.689
**Parity**		
No child (ref)	Ref.	Ref.
Had at least 1 child	1.15 (0.79-1.67)	.472
**Type of place of residence**		
Urban	1.32 (1.06-1.63)	.012
Rural(ref)	Ref.	Ref.
**Occupation**		
Not working(ref)	Ref.	Ref.
Professional	2.17 (1.48-3.18)	<.001
Agricultural	1.39 (1.04-1.85)	.024
Household and domestic	5.31[ 3.64-7.76)	<.001
**Manual**	2.78 (2.08-3.72)	<.001
**Wealth index status**		
Poorest (Ref.)	Ref.	Ref.
Poor	1.17 (0.90-1.51)	.241
Middle	1.05 (0.81-1.36)	.690
Rich	0.79 (0.59-1.05)	.100
Richest	0.68 (0.49-0.96)	.028
Own a mobile telephone	1.73 (1.45-2.08)	<.001
Husband/partner drinks alcohol	1.46 (1.24-1.71)	<.001
Respondent afraid of husband most of the time	3.16 (2.64-3.79)	<.001
Husband jealous if respondent talks with other men	0.89 (0.73-1.09)	.257
Husband accuses respondent of unfaithfulness	1.58 (1.31-1.89)	<.001
Husband/partner does not permit respondent to meet female friends	2.04 (1.66-2.50)	<.001
Husband/partner tries to limit respondent contact with family	1.26 (1.01-1.58)	.044
Husband /partner insists on knowing where the respondent is	1.03 (0.86-1.24)	.714

## Discussion

This study sought to determine interpersonal and socio-demographic factors associated with divorce among women of reproductive age using Tanzania 2015-16 TDHS-MIS data. The findings found that 13.3% of ever-married women were divorced or separated. Tanzania has a high rate of marriage, which is surprising given the African culture. This study aligns with statistics from 33 Sub-Saharan African nations, where 25% of first marriages end in divorce, ranging from 6.9% in Mali to 47.1% in Congo [6]. Yet, the divorce rate in this research is lower than the study done in Ethiopia, which found that 25% of women were divorced [24]. The difference in divorce rate could be caused by setting and social cultural aspects related to marriage. The study revealed that having at least one child was associated with a divorce. This contradicts other studies conducted elsewhere [25,42]. Furthermore, this study’s findings revealed a significant relationship between divorce and professional working women, which can be positively associated to either woman´s empowerment and which is related to a high educational level and independence. These findings corroborate with other study findings conducted elsewhere [19,24,47]. This study discovered that a lower age at first sex encounter is connected with divorce. Women who were under 15 years old at the time of their first marriage were more likely to divorce than those who were 18 years or older. This finding is consistent with other investigations [24,26,48]. The reason for this could be the difference in cognitive maturity level and preparation for marriage, since women who marry before the age of 15 have limited knowledge and plan for their marriage as compared to those who get married at age 18 years or more. Because of their immaturity, lack of preparation, and loss of interest, those girls are unable to plan and manage their families. Consequently, they are more likely to end up in divorce [38,48,27]. Furthermore, it has been shown that in most cases of early marriage, the girls were routinely subjected to subordination and violence by their husbands. This could indicate that the lower age at first sexual experience leads to early marriage and, as a result, divorce because some of them are young and unprepared for marriage.

The study discovered that increased marriage duration and senior age are connected with divorce. This contradicts Indonesian study findings, which found that married time is becoming less dependable predictors of marital stability [49]. The observed discrepancy could be ascribed to changes in the study's environment. Similarly, the present study revealed that residing in urban areas is associated with divorce. This finding corroborates other studies conducted elsewhere [6]. The observed difference between rural and urban locations could be explained by the fact that married women in rural areas accept marital problems better than women in urban areas, because women in rural areas rely more on their husbands financially. Furthermore, living in metropolitan regions with many temptations and moral disintegration could be grounds for a substantial correlation between urban locations and divorce. Additionally, the present study revealed the association between divorce and owning a mobile phone. Owning a mobile phone can simplify communication between with extramarital partners among married women. Thus, owning a phone can make extra marital communication easy, hence when caught might lead to infidelity accusations that might end up in divorce. This is in the same line with another study conducted elsewhere [47] where it was revealed that having a phone and access to internet is associated with divorce. The study further found that the widespread use of information and communication technology (ICT) can have detrimental impacts on relationships, leading to divorce. It has been documented that personal usage of ICT undermines both parenting and romantic relationships in couples. Furthermore, it was discovered that broadband internet habits and penetration have a considerable impact on the tendency for divorce [23,36]. The present study further revealed the association between husband/partner drinking alcohol and divorce. Also, drinking alcohol can lead to intimate partner violence and being irresponsible partner, something that might end up in divorce. IPV is one of the primary reasons claimed by couples seeking divorce [8,24,27]. Similarly, the current study demonstrated a link between being fearful of a husband/partner most of the time and divorce. Being terrified of your boyfriend or husband can indicate the prevalence of intimate partner abuse, which can lead to divorce [8,24].

This study revealed an association between husband/partner being jealous and divorce. When relationship jealousy is excessive, it can escalate to intimate partner violence and, subsequently, divorce [8, 24]. Furthermore, the present study revealed an association between being accused by a husband/partner for unfaithfulness and divorce. Our findings are in the same line with study findings conducted elsewhere [8,22]. The study showed an association between being restricted by husband/partner to meet female friends and divorce. It should be further noted that being restricted by husband/partner to meet female friends is one of the gender based violence- and when women become aware of that they might request divorce from their partners [8,24]. Likewise, the present study revealed an association between being limited by husband/partner to contact family and divorce. Again it should be noted that being limited by partner/husband to contact family is one of the gender based violence that women face in relationships and this might end to divorce [8,24,32,47]. Divorce is also viewed as an escape for women who have experienced various forms of intimate partner violence. These results are similar to other research conducted elsewhere [24,32]. Similarly, the current study found a substantial link between husband/partner's insistence on knowing where the respondent was and divorce. Again, insisting on knowing where the partner is can symbolize the jealous and controlling personality of some male partners, and when this goes too far, it can lead to intimate partner violence and subsequently to divorce [8,24].

**Limitations of the study:** however, this study has a few limitations: it was a cross-sectional study, thus it cannot prove the chronological order of divorce and its determinants. Regardless of its drawbacks, this study offers several strengths. One of the study's strengths is the utilization of nationally representative data with a high sample size. This could lead to a better prediction of parameters. Another merit of this study is that it is the first national-level effort to identify predictors of divorce among women of reproductive age that can be generalized.

## Conclusion

Several interpersonal and socio-demographic factors are linked to divorced women of reproductive age in Tanzania. Family life education is required to prevent divorce in marriages. Additionally, couples planning to marry should undergo adequate counseling on how to manage marital relationships and conflicts and live harmoniously.

### What is known about this topic

- Rates of divorce are increasing globally;
- Divorce causes diverse problems to children.

### What this study adds

- Thirteen point three percent (13.3%) of ever-married women were divorced or separated;
- Owning a mobile phone is associated with divorce;
- Residing in an urban area is associated with divorce.
